# Genetically proxied intestinal microbiota and risk of bladder cancer

**DOI:** 10.1097/JS9.0000000000001019

**Published:** 2024-01-04

**Authors:** Fuxun Zhang, Zhen Yao, Bo Zhang

**Affiliations:** Department of Urology, Tangdu Hospital, Air Force Medical University, Xi’an, Shaanxi, People’s Republic of China

*Dear Editor,*

The role of the gut microbiota in the pathophysiology, prognosis, and nutritional intervention of colorectal cancer has been well reviewed and summed up by Martínez-Montoro and colleagues at the *International Journal of Surgery* ^1^. This review enlightens us greatly. Actually, it has been established that intestinal microbiota is an important factor mediating environmental influence on human health and various diseases, not merely on gastrointestinal diseases^2^. Bladder cancer (BC) is prevalent globally and causes a significant public health challenge in the world. As the tenth most common malignancy, BC accounts for approximately 570 000 new cases and 213 000 deaths worldwide in 2020^3^. However, the causal relationship between gut microbiota and BC is less investigated. Thus, we performed this two-sample Mendelian randomization (MR) study to investigate the causal association between gut microbiota and BC.

MR analysis is a novel epidemiological method using single-nucleotide polymorphisms (SNPs) as instrumental variables (IVs) to replace the exposures and outcomes, which has been widely applied for etiological inferences for avoiding confounding factors and reverse causality. Summary statistics of human gut microbiota were obtained from the MiBioGen consortium^4^. Meanwhile, genome-wide association study (GWAS) summary statistics of BC were retrieved from FinnGen (https://r9.finngen.fi/) This study included 2053 BC cases and 307082 controls in the primary analyses. In addition, we excluded the cancer cases in other organs in controls, and then 287 137 controls were left as sensitivity analyses. All the participants were from Finns with European descent. Therefore, ethical approval is not needed in this study.

To obtain adequate IVs and increase the statistical power, IVs were filtered from the identified SNPs at a genome-wide statistical significance of *P*<1×10^−5^. The left SNPs were further pruned if the linkage disequilibrium *r* ^2^ were ≥0.01 at a window size of 10 000 kb. SNPs with minor allele frequency (MAF) <0.01 are generally accepted as rare SNPs, which have limited impact on the traits. Therefore, only SNPs with MAF ≥0.01 were reserved. Six methods were used to investigate the effects of gut microbiota on ED, including inverse variance weighting (IVW), MR-Egger, weighted median, maximum likelihood (ML), MR robust adjusted profile score (MR.RAPS), and MR pleiotropy residual sum and outlier (MR-PRESSO). Cochran *Q* test was applied to assess the heterogeneity of instrumental variables. *Q* statistics with *P* <0.05 indicated the presence of heterogeneity, and the random-effects IVW method was used to generate more conservative but robust estimates. To assess the horizontal pleiotropy, the MR-Egger intercept term and global test from MR-PRESSO estimator were used. The strength of SNPs was quantified via calculating *F*-statistics of each bacterial taxon as previously reported^5^. The *F*-statistic greater than 10 indicated less likelihood of weak instrumental bias. All statistical analyses were conducted using R 4.0.3.

As shown by the IVW estimator, *Lachnospiraceae* UCG004 [odds ratio (OR): 1.42], *Desulfovibrionales* (Order) (OR: 1.48), *Eubacterium ruminantium* group (OR: 1.33), *Olsenella* (OR: 1.24), *Ruminococcaceae* UCG002 (OR: 1.42), *Ruminococcaceae* UCG005 (OR: 1.44), and *Ruminococcaceae* UCG013 (OR: 1.60) were found to be causally associated with BC (All *P* < 0.05). Meanwhile, *Bacteroidetes* (Phylum) (OR: 0.61), *Eubacterium brachy* group (OR: 0.80), *Ruminococcaceae* UCG004 (OR: 0.73), *Rikenellaceae* (Family) (OR: 0.67), *Lachnospiraceae* ND3007 group (OR: 0.47), and *Adlercreutzia* (OR: 0.73) revealed protective effects against BC (all *P*<0.05) (Fig. 1). The effect sizes and directions remained consistent with other five methods and sensitivity analyses (Fig. 1). No heterogeneity and pleiotropy were detected by Cochran’s *Q* test, MR-Egger and global test (all *P*>0.05) (Supplementary Figs S1–S6, Supplemental Digital Content 1, http://links.lww.com/JS9/B636, Supplemental Digital Content 2, http://links.lww.com/JS9/B637, Supplemental Digital Content 3, http://links.lww.com/JS9/B638, Supplemental Digital Content 4, http://links.lww.com/JS9/B639, Supplemental Digital Content 5, http://links.lww.com/JS9/B640, Supplemental Digital Content 6, http://links.lww.com/JS9/B641) (Supplementary Table S1, Supplemental Digital Content 7, http://links.lww.com/JS9/B642 and Table S2, Supplemental Digital Content 8, http://links.lww.com/JS9/B643).

**Figure 1:**
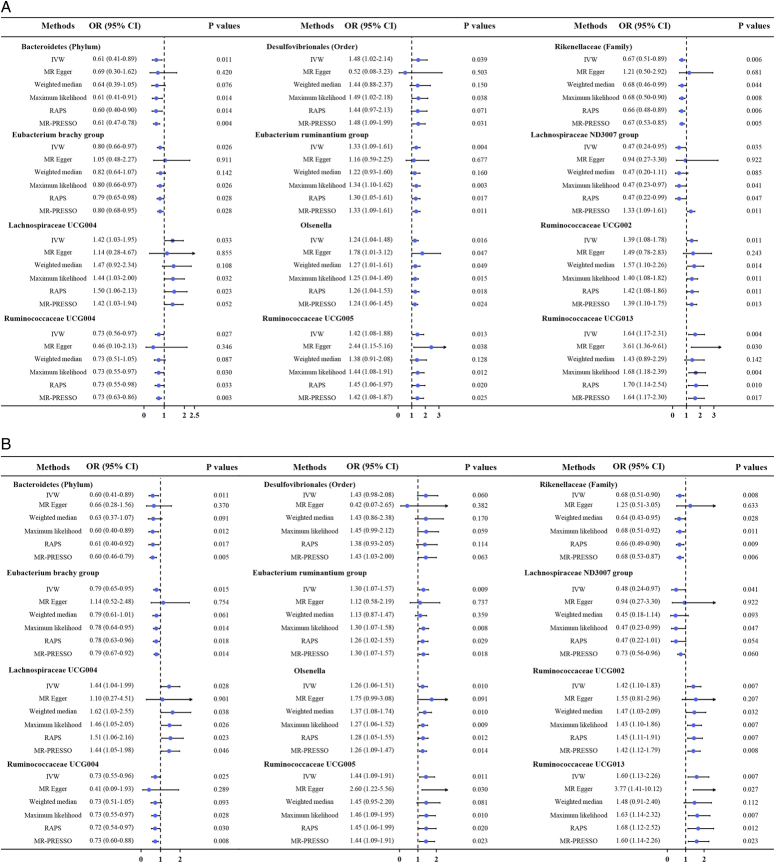
The results of MR estimating the causal association between intestinal microbiota and bladder cancer (A). The results of MR estimating the causal association between intestinal microbiota and bladder cancer with controls excluded all cancers (B). CI, confidence interval; IVW, inverse variance weighted; MR, Mendelian randomization; OR, odds ratio; PRESSO, pleiotropy residual sum and outlier; RAPS, robust adjusted profile score.

In this two-sample MR analysis, we found that genetically proxied *Lachnospiraceae* UCG004, *Desulfovibrionales* (Order), *Eubacterium ruminantium* group, *Olsenella*, *Ruminococcaceae* UCG002, *Ruminococcaceae* UCG005, and *Ruminococcaceae* UCG013 had causal effects on BC. Meanwhile, *Bacteroidetes* (Phylum), *Eubacterium brachy group*, *Ruminococcaceae* UCG004, *Rikenellaceae* (Family), *Lachnospiraceae* ND3007 group, *Adlercreutzia*, and one unknown genus demonstrated protective effects against BC. Further studies are needed to investigate the molecular mechanisms linking intestinal microbiota and BC.

## Ethical approval

Ethical approval is not needed in this study.

## Consent

Not applicable.

## Sources of funding

This work was supported by the National Natural Science Foundation of China (No. 81872077).

## Author contribution

F.Z., Z.Y., and B.Z.: conception and design; B.Z.: administrative support; F.Z. and Z.Y.: provision of study materials or patients, collection and assembly of data, data analysis and interpretation, and manuscript writing; F.Z., Z.Y., and B.Z.: final approval of the manuscript.

## Conflicts of interest disclosure

All authors report no conflicts of interest in this work.

## Research registration unique identifying number (UIN)

Not applicable.

## Guarantor

Bo Zhang.

## Data availability statement

The datasets analyzed during the current study are available from the corresponding author on reasonable request.

## Provenance and peer review

Our paper was not invited.

## Supplementary Material

SUPPLEMENTARY MATERIAL

## Supplementary Material

**Figure:**
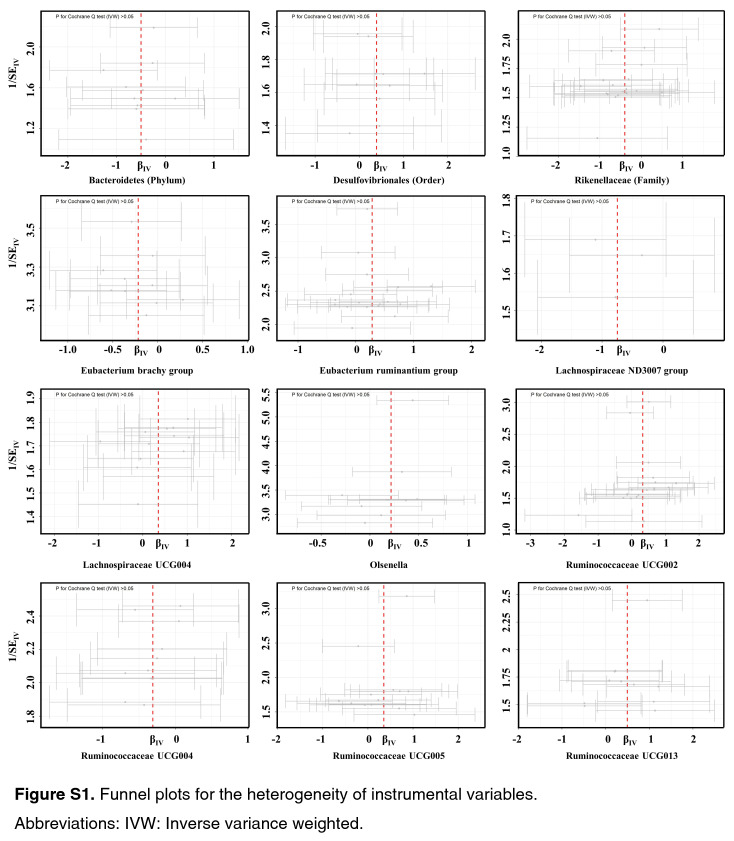


**Figure:**
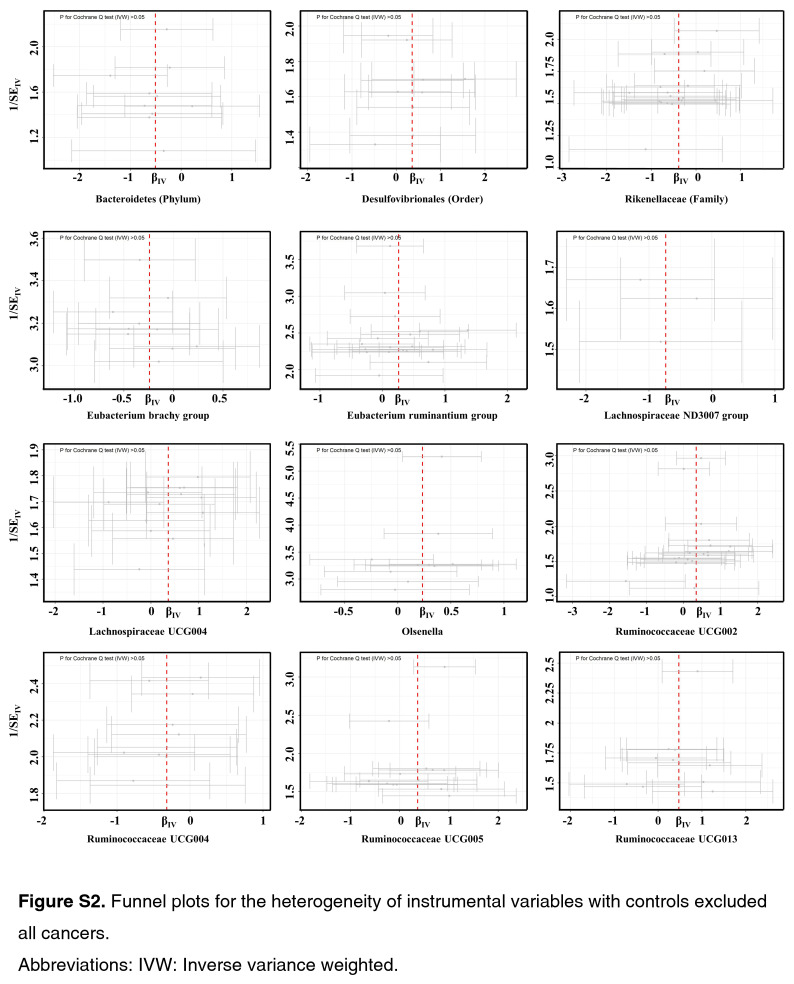


**Figure:**
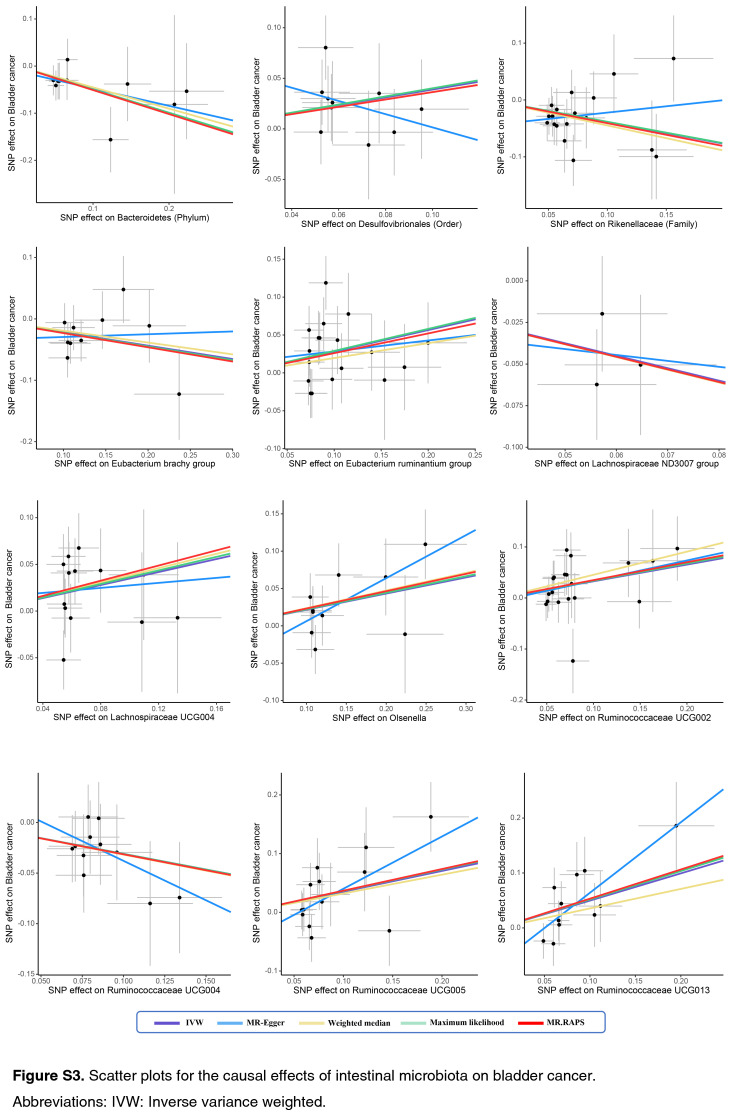


**Figure:**
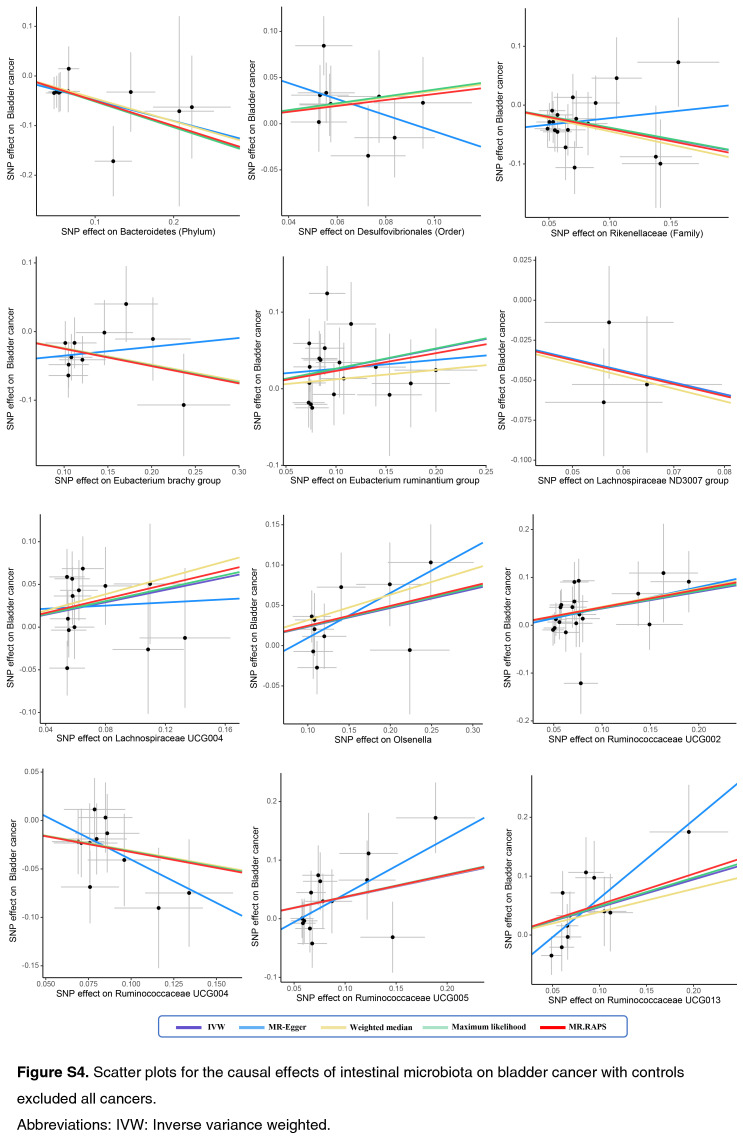


**Figure:**
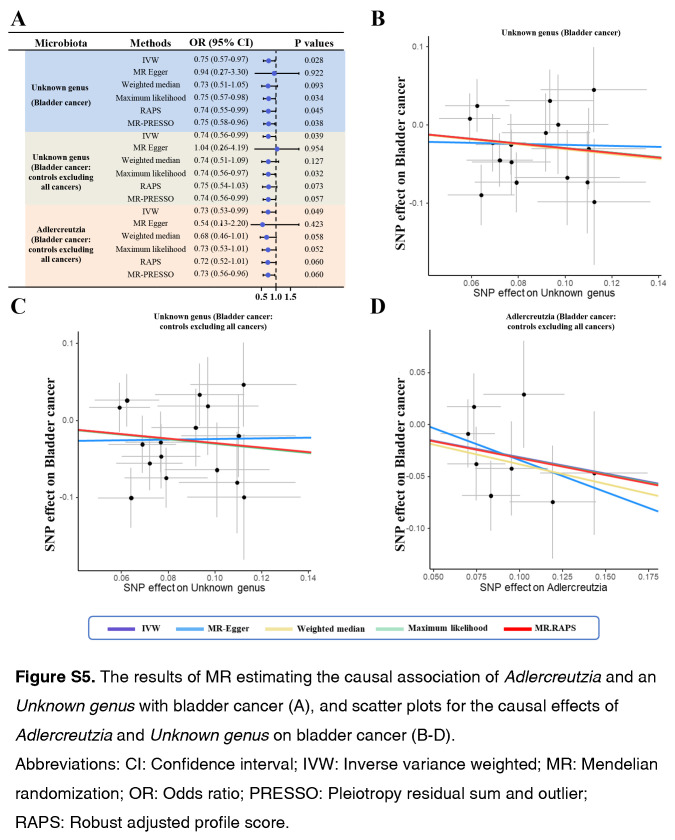


**Figure:**
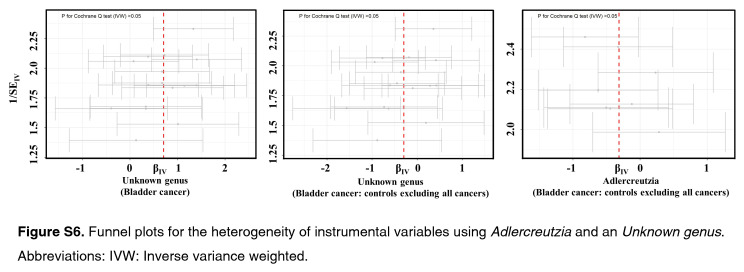


## References

[1] Martínez-Montoro JI Martínez-Sánchez MA Balaguer-Román A . Dietary modulation of gut microbiota in patients with colorectal cancer undergoing surgery: a review. Int J Surg 2022;104:106751. 35803517 10.1016/j.ijsu.2022.106751

[2] Lynch SV Pedersen O . The human intestinal microbiome in health and disease. N Engl J Med 2016;375:2369–2379. 27974040 10.1056/NEJMra1600266

[3] Patel VG Oh WK Galsky MD . Treatment of muscle-invasive and advanced bladder cancer in 2020. CA Cancer J Clin 2020;70:404–423. 32767764 10.3322/caac.21631

[4] Kurilshikov A Medina-Gomez C Bacigalupe R . Large-scale association analyses identify host factors influencing human gut microbiome composition. Nat Genet 2021;53:156–165. 33462485 10.1038/s41588-020-00763-1 PMC8515199

[5] XiongY ZhangF ZhangY . Insights into modifiable risk factors of erectile dysfunction, a wide-angled Mendelian Randomization study. J Adv Res 2023;S2090-1232:00147–00149.10.1016/j.jare.2023.05.00837236543

