# Supplementary material for: Genetically proxied intestinal microbiota and risk of bladder cancer
Source: Int J Surg. 2024 Jan 4;110(3):1857–9. doi: 10.1097/JS9.0000000000001019 (PMC10942171; doi:10.1097/JS9.0000000000001019)
Supplement: SUPPLEMENTARY MATERIAL [file js9-110-1857-s007.docx]

Table 1. Detailed results regarding the strength and pleiotropy of instrumental variables for bladder cancer.

| **Microbiota** | **Number of SNPs** | **F-statistics** | **Bladder cancer** | | | **Bladder cancer (controls excluding all cancers)** | | |
| --- | --- | --- | --- | --- | --- | --- | --- | --- |
|  |  |  | **P for Global test** | **Intercept for Egger regression** | **P for Egger** | **P for Global test** | **Intercept for Egger regression** | **P for Egger** |
| ***Bacteroidetes (Phylum)*** | 10 | 21.98 | 0.917 | -0.010763057 | 0.743 | 0.898 | -0.0072 | 0.827 |
| ***Desulfovibrionales (Order)*** | 10 | 21.52 | 0.772 | 0.066853813 | 0.286 | 0.663 | 0.0788 | 0.219 |
| ***Rikenellaceae (Family)*** | 18 | 21.47 | 0.773 | -0.042633087 | 0.189 | 0.803 | -0.0443 | 0.177 |
| ***Adlercreutzia*** | - | - | - | - | - | 0.629 | 0.0274 | 0.680 |
| ***Eubacterium brachy group*** | 10 | 20.68 | 0.715 | -0.034073703 | 0.511 | 0.773 | -0.0480 | 0.365 |
| ***Eubacterium ruminantium group*** | 18 | 21.37 | 0.393 | 0.01388457 | 0.677 | 0.389 | 0.0144 | 0.664 |
| ***Lachnospiraceae ND3007 group*** | 3 | 20.94 | 0.393 | -0.023124659 | 0.958 | 0.629 | 0.0008 | 0.999 |
| ***Lachnospiraceae UCG004*** | 13 | 21.24 | 0.486 | 0.014091035 | 0.764 | 0.495 | 0.0178 | 0.706 |
| ***Olsenella*** | 10 | 21.47 | 0.601 | -0.051323079 | 0.223 | 0.712 | -0.0462 | 0.274 |
| ***Ruminococcaceae UCG002*** | 19 | 21.47 | 0.648 | -0.00579333 | 0.820 | 0.697 | -0.0072 | 0.781 |
| ***Ruminococcaceae UCG004*** | 11 | 21.57 | 0.978 | 0.040001985 | 0.558 | 0.925 | 0.0489 | 0.480 |
| ***Ruminococcaceae UCG005*** | 14 | 21.21 | 0.481 | -0.048903757 | 0.156 | 0.433 | -0.0536 | 0.127 |
| ***Ruminococcaceae UCG013*** | 11 | 22.01 | 0.476 | -0.064493993 | 0.126 | 0.492 | -0.0701 | 0.103 |
| ***Unknown genus*** | 16 | 21.87 | 0.521 | -0.018874903 | 0.721 | 0.354 | -0.0278 | 0.635 |

Abbreviations: MR: Mendelian randomization; SNPs: Single nucleotide polymorphisms.
