# Supplementary material for: Genetically proxied intestinal microbiota and risk of bladder cancer
Source: Int J Surg. 2024 Jan 4;110(3):1857–9. doi: 10.1097/JS9.0000000000001019 (PMC10942171; doi:10.1097/JS9.0000000000001019)
Supplement: SUPPLEMENTARY MATERIAL [file js9-110-1857-s008.docx]

Table S1. The detailed results for heterogeneity of instrumental variables.

| **Microbiota** | **Methods** | **Bladder cancer** | | **Bladder cancer (controls excluding all cancers)** | |
| --- | --- | --- | --- | --- | --- |
|  |  | **Q values** | **P values** | **Q values** | **P values** |
| ***Bacteroidetes (Phylum)*** | MR-Egger | 3.704 | 0.883 | 4.206 | 0.838 |
|  | IVW | 3.819 | 0.923 | 4.256 | 0.894 |
|  | ML | 3.714 | 0.929 | 4.133 | 0.902 |
| ***Desulfovibrionales (Order)*** | MR-Egger | 4.541 | 0.805 | 5.302 | 0.725 |
|  | IVW | 5.849 | 0.755 | 7.082 | 0.629 |
|  | ML | 5.732 | 0.766 | 6.965 | 0.641 |
| ***Rikenellaceae (Family)*** | MR-Egger | 10.311 | 0.850 | 10.311 | 0.850 |
|  | IVW | 12.301 | 0.782 | 12.301 | 0.782 |
|  | ML | 12.071 | 0.796 | 12.071 | 0.796 |
| ***Adlercreutzia*** | MR-Egger | - | - | 5.191 | 0.520 |
|  | IVW | - | - | 5.378 | 0.614 |
|  | ML | - | - | 5.259 | 0.628 |
| ***Eubacterium brachy group*** | MR-Egger | 6.043 | 0.642 | 18.137 | 0.316 |
|  | IVW | 6.517 | 0.687 | 18.360 | 0.366 |
|  | ML | 6.367 | 0.703 | 18.042 | 0.386 |
| ***Eubacterium ruminantium group*** | MR-Egger | 18.674 | 0.286 | 4.864 | 0.772 |
|  | IVW | 18.884 | 0.335 | 5.787 | 0.761 |
|  | ML | 18.492 | 0.358 | 5.629 | 0.776 |
| ***Lachnospiraceae ND3007 group*** | MR-Egger | 0.809 | 0.368 | 1.104 | 0.293 |
|  | IVW | 0.813 | 0.666 | 1.104 | 0.576 |
|  | ML | 0.758 | 0.685 | 1.033 | 0.597 |
| ***Lachnospiraceae UCG004*** | MR-Egger | 11.532 | 0.400 | 11.427 | 0.408 |
|  | IVW | 11.631 | 0.476 | 11.582 | 0.480 |
|  | ML | 11.461 | 0.490 | 11.405 | 0.495 |
| ***Olsenella*** | MR-Egger | 5.381 | 0.716 | 5.082 | 0.749 |
|  | IVW | 7.130 | 0.624 | 6.463 | 0.693 |
|  | ML | 6.925 | 0.645 | 6.262 | 0.713 |
| ***Ruminococcaceae UCG002*** | MR-Egger | 15.467 | 0.562 | 14.864 | 0.605 |
|  | IVW | 15.520 | 0.626 | 14.944 | 0.666 |
|  | ML | 15.314 | 0.640 | 14.728 | 0.681 |
| ***Ruminococcaceae UCG004*** | MR-Egger | 2.853 | 0.970 | 4.097 | 0.905 |
|  | IVW | 3.224 | 0.976 | 4.639 | 0.914 |
|  | ML | 3.159 | 0.977 | 4.541 | 0.920 |
| ***Ruminococcaceae UCG005*** | MR-Egger | 10.055 | 0.611 | 10.001 | 0.616 |
|  | IVW | 12.350 | 0.499 | 12.689 | 0.472 |
|  | ML | 12.028 | 0.525 | 12.335 | 0.500 |
| ***Ruminococcaceae UCG013*** | MR-Egger | 6.977 | 0.639 | 6.673 | 0.671 |
|  | IVW | 9.816 | 0.457 | 9.964 | 0.444 |
|  | ML | 9.492 | 0.486 | 9.677 | 0.469 |
| ***Unknown genus*** | MR-Egger | 13.992 | 0.450 | 16.719 | 0.271 |
|  | IVW | 14.125 | 0.516 | 17.001 | 0.319 |
|  | ML | 13.935 | 0.531 | 16.765 | 0.333 |

Abbreviations: IVW: Inverse variance weighting; ML: Maximum likelihood; MR: Mendelian randomization.
